# White Matter Disease Correlates with Lexical Retrieval Deficits in Primary Progressive Aphasia

**DOI:** 10.3389/fneur.2013.00212

**Published:** 2013-12-27

**Authors:** John P. Powers, Corey T. McMillan, Caroline C. Brun, Paul A. Yushkevich, Hui Zhang, James C. Gee, Murray Grossman

**Affiliations:** ^1^Department of Neurology, Penn Frontotemporal Degeneration Center, University of Pennsylvania Perelman School of Medicine, Philadelphia, PA, USA; ^2^Penn Image Computing and Science Laboratory, Department of Radiology, University of Pennsylvania Perelman School of Medicine, Philadelphia, PA, USA; ^3^Department of Computer Science, Centre for Medical Image Computing, University College London, London, UK

**Keywords:** frontotemporal dementia, primary progressive aphasia, diffusion-weighted MRI, magnetic resonance imaging, neuropsychology

## Abstract

**Objective:** To relate fractional anisotropy (FA) changes associated with the semantic and logopenic variants of primary progressive aphasia (PPA) to measures of lexical retrieval.

**Methods:** We collected neuropsychological testing, volumetric magnetic resonance imaging, and diffusion-weighted imaging on semantic variant PPA (svPPA) (*n* = 11) and logopenic variant PPA (lvPPA) (*n* = 13) patients diagnosed using published criteria. We also acquired neuroimaging data on a group of demographically comparable healthy seniors (*n* = 34). FA was calculated and analyzed using a white matter (WM) tract-specific analysis approach. This approach utilizes anatomically guided data reduction to increase sensitivity and localizes results within canonically defined tracts. We used non-parametric, cluster-based statistical analysis to relate language performance to FA and determine regions of reduced FA in patients.

**Results:** We found widespread FA reductions in WM for both variants of PPA. FA was related to both confrontation naming and category naming fluency performance in left uncinate fasciculus and corpus callosum in svPPA and left superior and inferior longitudinal fasciculi in lvPPA.

**Conclusion:** SvPPA and lvPPA are associated with distinct disruptions of a large-scale network implicated in lexical retrieval, and the WM disease in each phenotype may contribute to language impairments including lexical retrieval.

## Introduction

Primary progressive aphasia (PPA) is a clinical syndrome characterized by progressive loss of language most commonly due to Alzheimer’s disease (AD) or frontotemporal lobar degeneration (FTLD). Lexical retrieval difficulty is a feature of two variants of PPA (1). Semantic variant PPA (svPPA) is characterized by deficits in single-word and object comprehension as well as poor confrontation naming (2). Logopenic variant PPA (lvPPA) is characterized by impaired single-word retrieval and impaired repetition of phrases and sentences (1). This study sought to determine whether impaired lexical retrieval in these two variants of PPA is due to common or distinct patterns of white matter (WM) disease.

Neuropathological and neuroimaging studies have identified patterns of gray matter (GM) atrophy in these PPA variants: svPPA is associated with left-lateralized GM atrophy of the anterior and ventral temporal lobe, while lvPPA is associated with atrophy of left posterior peri-Sylvian and inferior parietal regions (3–5). GM atrophy in these conditions may overlap in the posterolateral mid-temporal lobe (6), an area that is associated with lexical retrieval (7, 8). The underlying pathology can also impact WM (9, 10), and a growing number of studies have investigated WM disease in PPA using diffusion tensor imaging (DTI) (11–15). However, the relationship between WM disease and clinical features such as lexical retrieval remains unclear.

We used a tract-specific analysis (TSA) technique (16) to assess how changes in fractional anisotropy (FA) associated with svPPA and lvPPA contribute to lexical retrieval impairments. TSA involves anatomically guided data reduction in 11 major WM tracts. We chose TSA over other WM analysis techniques to utilize a user-independent approach. Additionally, TSA offers increased sensitivity to detect WM changes by minimizing potential confounds introduced through the smoothing and suboptimal registration processes of most analytic diffusion tensor (DT) approaches. We assessed semantically guided category naming fluency and confrontation naming in svPPA and lvPPA patients and related these measures to FA. We hypothesized that lexical retrieval would be related to interruption of distinct WM components of a large-scale neural network subserving this process in svPPA and lvPPA.

## Materials and Methods

### Participants

We recruited 24 patients from the Penn Frontotemporal Degeneration Center that met diagnostic criteria for svPPA (*n* = 11) or lvPPA (*n* = 13) (1). All svPPA cases were apparent sporadic as mutations were not present in the three major genes associated with FTLD (*C9orf72*, *MAPT*, *GRN*). No mutations were present in these genes in the lvPPA patients who had been screened, but genetic screening was not performed for many lvPPA patients due to the common association of this phenotype with AD pathology. All patients underwent neuropsychological assessment, structural magnetic resonance imaging (MRI), and DTI. Exclusionary criteria included other neurological conditions such as stroke, head trauma, or hydrocephalus; other causes of dementia; medical conditions associated with cognitive difficulty; and primary psychiatric disorders. Some patients may have been taking a small dosage of a non-sedating anti-depressant (e.g., sertraline) or a low-potency non-sedating neuroleptic (e.g., quetiapine) as needed medically. A control group of 34 healthy seniors also underwent structural MRI and DTI. Control participants were comparable to patients for age (*F*(2,55) = 0.736; *p* = 0.484; Table 1), sex (χ^2^(2, *n* = 58) = 0.945; *p* = 0.623), and education (*F*(2,55) = 2.830; *p* = 0.068). Patient groups were also comparable for Mini-Mental State Examination (MMSE) performance (*t*(22) = 0.189; *p* = 0.852; Table 1) and disease duration at MRI (*t*(22) = −1.363; *p* = 0.187). We report group-level GM imaging differences in svPPA and lvPPA relative to controls in the Supplementary Material (Web Only Materials). Briefly, these results confirm characteristic distributions of GM disease for each patient group: svPPA showed atrophy in left anterior temporal lobe extending into posterior temporal regions, and lvPPA displayed atrophy in left peri-Sylvian cortex and superior temporal lobe (Figure S1 in Supplementary Material, Web Only Materials).

**Table 1:** **Mean (±SD) clinical and demographic features and neuropsychological performance**.

	lvPPA	svPPA	Controls
Age (years)	65.9 (7.5)	63.8 (7.4)	62.8 (7.9)
Sex (M/F)	6/7	4/7	18/16
Education (years)	14.6 (2.8)	17.2 (3.4)	15.3 (2.5)
MMSE (max. 30)	22.8 (3.9)	22.4 (6.5)	N/A
Disease duration at MRI (years)	2.7 (1.8)	3.9 (2.6)	N/A
Category naming fluency (*z*-score)	−2.3 (0.9)	−2.8 (0.8)	N/A
Boston Naming test (*z*-score)	−5.6 (3.8)	−13.2 (3.4)	N/A
Highest Backwards Digit Span (*z*-score)	−1.1 (0.7)^a^	−1.0 (1.3)	N/A
Rey recall total (*z*-score)	−1.6 (1.4)^a^	−0.8 (1.0)^a^	N/A

*^a^Score not available for one subject in the group*.

### Standard protocol approvals, registrations, and patient consents

Written informed consent was obtained for all participants using a protocol approved by the University of Pennsylvania Institutional Review Board.

### Neuropsychological assessment

We assessed semantically guided category naming fluency [Animals; Ref. (17)] by asking patients to orally name as many different words as possible belonging to a target semantic category (animals) in a 60-s period. Total score was calculated as the number of unique words meeting the target category criterion.

An abbreviated version of the Boston Naming Test [BNT; Ref.(18)] was used to assess confrontation naming. Patients were asked to orally name each test stimulus (*n* = 30). Stimuli consisted of black-and-white line drawings. Target names were equally distributed between high frequency, mid-frequency, and low-frequency items. Patients were given as much time as needed to respond. The number of stimuli that were correctly identified was counted as the total score.

Patients were also tested for highest correct backwards digit span and recall of the Rey figure to verify normal performance in cognitive domains outside of language. We report patient *z*-scores for neuropsychological measures generated relative to a cohort of demographically comparable healthy seniors (*p* > 0.09 for sex, age, and education; *n* = 24; Table 1).

### Imaging data acquisition

Imaging was acquired on the same day as neuropsychological testing for 16 of the 24 patients, and overall, was acquired within an average of 2 months (mean = 1.5 ± 2.9 months) from neuropsychological testing. Diffusion-weighted images (DWI) were acquired on a Siemens 3.0 T Trio scanner with an 8-channel coil using a single-shot, spin-echo, diffusion-weighted echo planar imaging sequence (FOV = 245 mm; matrix size = 112 × 112; number of slices = 57; voxel size = 2.2 mm isotropic; TR = 6700 ms; TE = 85 ms; fat saturation). In total, 31 volumes were acquired per subject, one without diffusion weighting (*b* = 0 s/mm^2^) and 30 with diffusion weighting (*b* = 1000 s/mm^2^) along 30 non-collinear directions. A structural T1-weighted 3-dimensional spoiled gradient-echo sequence was also collected in the same session with TR = 1620 ms, TE = 3 ms, flip angle = 15°, matrix = 192 × 256, slice thickness = 1 mm, and in-plane resolution = 1.0 mm × 1.0 mm.

### Preprocessing of diffusion-weighted images

Diffusion tensors were reconstructed from the DWI using Camino (19). After rigid transformation, tensor images were aligned to a healthy aging template through affine and diffeomorphic registration using the Diffusion Tensor Imaging ToolKit (DTI-TK)^1^ (20, 21). DTI-TK has been confirmed as a leading tool in DTI registration (22). The healthy aging template used in the registration is the same template that was used to generate the WM tract models as described below.

### Tract-specific analysis

**Figure 1:**
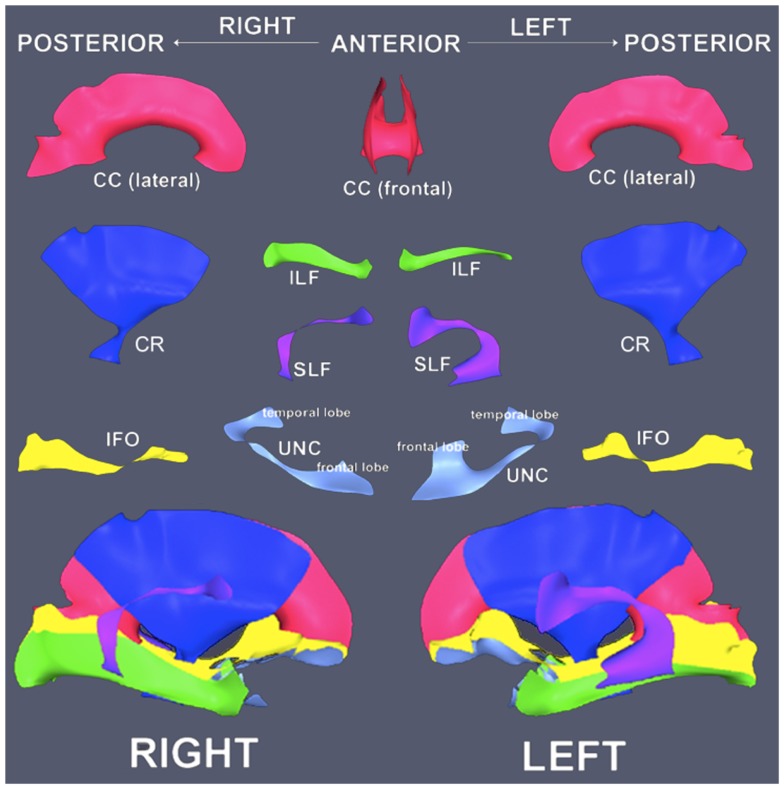
**Orientation diagram of white matter tract models**. Tract models are presented in corresponding arrangement to the visualizations of the results (top). Tract models are assembled in anatomical context (bottom). Note the UNC models are rotated in the isolated view relative to the anatomical arrangement to display the surface more effectively. CC, corpus callosum; CR, corona radiata; ILF, inferior longitudinal fasciculus; SLF, superior longitudinal fasciculus; IFO, inferior fronto-occipital fasciculus; UNC, uncinate fasciculus.

A detailed description of the TSA approach is available elsewhere (16). Briefly, 11 tracts were available for use with TSA: corpus callosum (CC) and bilateral inferior fronto-occipital fasciculus (IFO), inferior longitudinal fasciculus (ILF), superior longitudinal fasciculus (SLF), uncinate fasciculus (UNC), and corticofugal fibers including the corona radiata (CR). The tracts were defined from an aging DTI template generated from 51 subjects (21 males/30 females, mean age = 70.1 ± 4.0 years) from the publicly available IXI database^2^ (23). We chose a healthy aging template to match the demographic characteristics of our research population and to model diffusion pathways without severe disease-related alterations. Whole-brain tractography was run on the template using the Fiber Assignment by Continuous Tracking (FACT) algorithm in DTI-TK (24), and an established ROI-based fiber-tracking protocol was used to segment the tracts of interest (25). Each tract was then converted to a binary volume and modeled by a skeleton and its corresponding boundary using a continuous medial model (Figure 1). For each point in the skeleton, a line segment called a spoke connects the point to the closest point on the model’s boundary and establishes a depth coordinate system. Spokes are orthogonal to the model’s boundary, and no spokes in the model may intersect.

For each subject and each tract, maximum FA values were computed from the DT image along the spokes of the model and projected onto the corresponding point on the model’s surface. *T*-tests were used to compare each patient group to controls at each point on the model. Using a *t*-value height threshold corresponding to *p* < 0.005, non-parametric, cluster-based statistical analysis was run with 10,000 permutations. We applied familywise error (FWE) correction for multiple comparisons and report clusters surviving a size threshold equivalent to *p* < 0.05. Additionally, we ran regression analyses with language measures in tracts where clusters of significantly decreased FA were identified in patients relative to controls. Analyses were constrained to tracts with FA disruptions so impairments in lexical retrieval could be related to disease. Regression analyses were run with 10,000 permutations and used a *t*-value height threshold of *p* < 0.01. We applied a cluster size threshold of *p* < 0.05 with FWE correction to identify clusters where total score on BNT or Animals correlated with FA.

## Results

**Figure 2:**
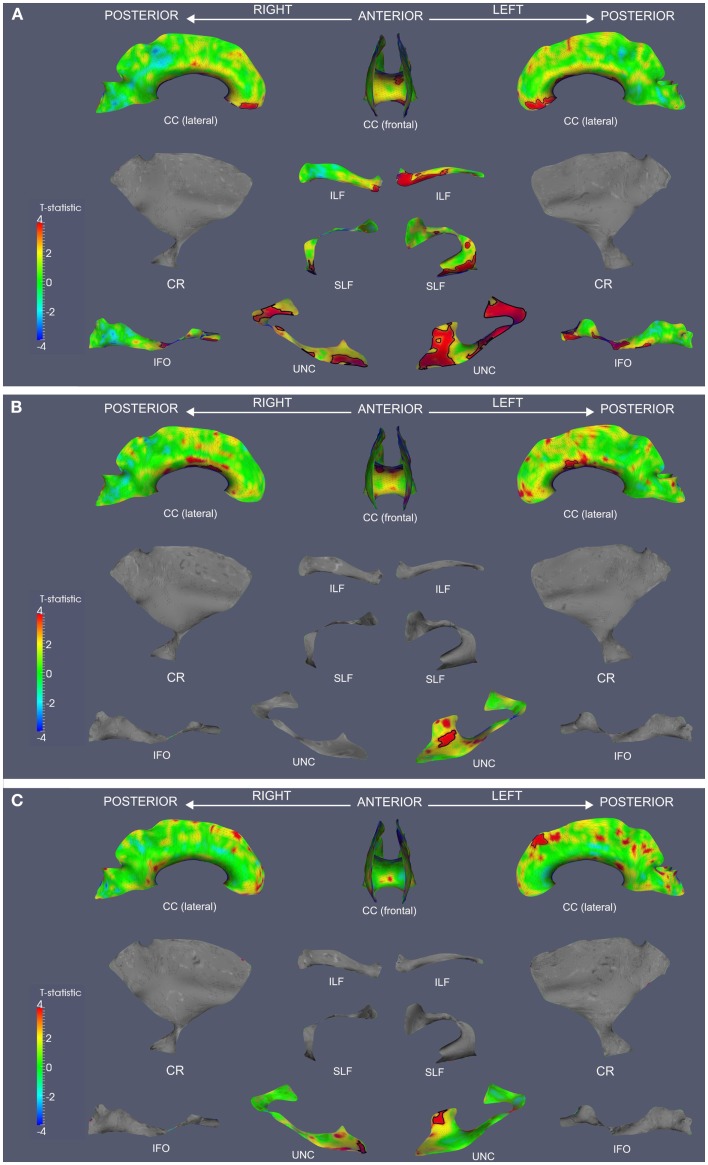
**Fractional anisotropy reductions and correlations with Animals and BNT in svPPA**. TSA analysis contrasting FA in svPPA to healthy seniors **(A)**. TSA regression analysis in svPPA correlating Animals total score and FA **(B)**. TSA regression analysis in svPPA correlating BNT total score and FA **(C)**. In all panels, red corresponds with higher *t*-values, and statistically significant clusters are outlined in black. CC, corpus callosum; CR, corona radiata; ILF, inferior longitudinal fasciculus; SLF, superior longitudinal fasciculus; IFO, inferior fronto-occipital fasciculus; UNC, uncinate fasciculus.

**Figure 3:**
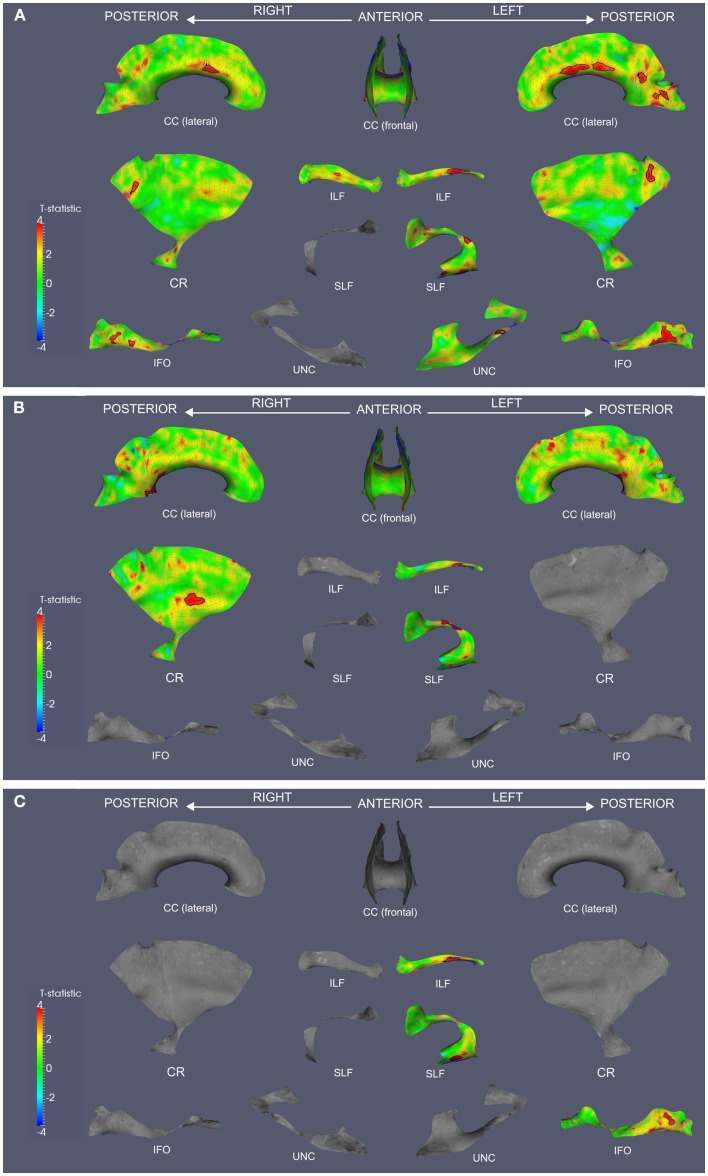
**Fractional anisotropy reductions and correlations with Animals and BNT in lvPPA**. TSA analysis contrasting FA in lvPPA to healthy seniors **(A)**. TSA regression analysis in lvPPA correlating Animals total score and FA **(B)**. TSA regression analysis in lvPPA correlating BNT total score and FA **(C)**. In all panels, red corresponds with higher *t*-values, and statistically significant clusters are outlined in black. CC, corpus callosum; CR, corona radiata; ILF, inferior longitudinal fasciculus; SLF, superior longitudinal fasciculus; IFO, inferior fronto-occipital fasciculus; UNC, uncinate fasciculus.

We observed widespread FA reductions in svPPA and lvPPA relative to controls. These results are illustrated in Figures 2A and 3A, and cluster features of all significant results are summarized in Table 2. Bilateral FA changes were present in both patient groups; however, areas of decreased FA were consistently more extensive in left-lateralized structures. Specifically, svPPA patients demonstrated reduced FA in bilateral ILF, IFO, SLF, and UNC as well as CC relative to controls. These changes were most apparent in anterior and ventral portions of these tracts. In lvPPA, decreases in FA were present in bilateral CR, ILF, and IFO in addition to CC; left SLF and left UNC tracts also demonstrated reduced FA.

**Table 2:** **Clusters of reduced fractional anisotropy and regressions relating fractional anisotropy to language measures**.

WM tract	Area (mm^2^)	*p*-Value
**svPPA < SENIORS**		
L UNC	520	0.001
	578	0.001
L ILF	939	0.001
	15	0.040
L IFO	545	0.001
	86	0.030
	353	0.001
	410	0.001
L SLF	419	0.001
	40	0.028
	26	0.048
CC	158	0.018
	296	0.007
	327	0.005
	162	0.017
R UNC	182	0.001
	130	0.001
	10	0.040
	12	0.035
R ILF	127	0.002
R IFO	110	0.006
	94	0.010
R SLF	35	0.012
	45	0.006
**lvPPA < SENIORS**		
L SLF	69	0.007
	28	0.037
	41	0.021
L ILF	227	0.001
L UNC	15	0.042
L IFO	449	0.001
L CR	93	0.017
CC	121	0.026
	86	0.042
	170	0.016
	100	0.033
	293	0.007
R CR	46	0.049
R IFO	63	0.019
	110	0.007
R ILF	27	0.026
**svPPA ANIMALS REGRESSION**		
L UNC	97	0.007
CC	174	0.030
	174	0.030
	159	0.037
**svPPA BNT REGRESSION**		
L UNC	58	0.021
R UNC	38	0.024
CC	212	0.019
**lvPPA ANIMALS REGRESSION**		
L SLF	77	0.023
L ILF	39	0.035
CC	415	0.018
R CR	147	0.032
**lvPPA BNT REGRESSION**		
L SLF	73	0.014
L ILF	120	0.007
L IFO	106	0.021

Both patient groups demonstrated impaired performance on Animals and BNT (Table 1). SvPPA performed worse on BNT relative to lvPPA (*t*(22) = 5.160; *p* = 0.001), but performance was comparable for Animals (*t*(22) = 1.407; *p* = 0.173). In svPPA, regression analysis related total score on Animals to FA changes in left UNC and CC (Figure 2B), and BNT total score also was related to changes in FA in UNC and CC (Figure 2C). In lvPPA, score on Animals was related to FA changes in left SLF, left ILF, right CR and CC (Figure 3B), and BNT score was associated with FA in left SLF, ILF, and IFO (Figure 3C).

## Discussion

Lexical retrieval is impaired in both svPPA and lvPPA. In this study, a TSA approach to DTI demonstrated that distinct distributions of FA changes contribute to this deficit in svPPA and lvPPA. These findings suggest that the clinical deficits associated with PPA reflect the breakdown of a large-scale neural network that ultimately involves the coordination of multiple brain regions contributing to these complex language tasks. We discuss these findings in more detail below.

In svPPA, impairments in semantically guided category naming fluency and confrontation naming were both related to WM changes in the frontal portion of the left UNC. This WM tract carries projections between the anterior and ventral temporal lobe and GM regions in the frontal lobe. Anterior and ventral temporal lobe structures are thought to contribute to word meaning (26, 27), while frontal lobe structures are thought to play a role in lexical selection and retrieval (28, 29). These are two of the major components shared by category naming fluency and confrontation naming, and this may explain in part why difficulty on these tasks is so prominent in svPPA. Moreover, implication of right UNC is consistent with the bilateral nature of selection and retrieval processes in PPA, even when the material is verbal (30). CC may contribute by helping to integrate left and right hemispheric functioning. Agosta et al. assessed BNT score and mean diffusivity (MD) of CC and various left-lateralized fiber tracts in patients with semantic dementia but found no significant correlation (11). As discussed below, the higher sensitivity afforded by TSA may contribute to the detection of correlations in the present study that had not been previously observed.

In lvPPA, by comparison, reduced WM integrity related confrontation naming to left SLF, ILF, and IFO. SLF and IFO project from frontal regions to the posterior temporal region, an area that has been associated with lexical representations (7). We also found that category naming fluency deficits in lvPPA correlated with disease affecting left SLF, left ILF, CC, and right CR. Disease in the ILF may contribute to lexical retrieval, and disease in the dorsal stream in SLF may play a role in the phonological speech errors found in lvPPA (1). Naming is a complex process involving semantic knowledge, visuospatial information, and lexical search; these results suggest that impairment in distinct processes involved in confrontation naming and category naming fluency may associate with distinct distributions of damage to a large-scale lexical retrieval network (8). Taken together with the observations in svPPA, these findings are consistent with the hypothesis that lexical retrieval depends on a neural network involving anterior-ventral temporal, posterior temporal, and lateral prefrontal GM regions and the WM projections between them. Both svPPA and lvPPA appear to have overlapping GM disease in the posterolateral temporal lobe that may contribute to impaired lexical retrieval, but distinct patterns of WM interruption of this network in svPPA and lvPPA also play a role in lexical retrieval difficulty.

Previous work has implicated both dorsal and ventral streams in language (31, 32). Specifically, syntactic and phonological processing has been more associated with a dorsal stream mediated by the SLF. While less work has investigated the role of the ventral stream mediated by IFO, it is thought to be more involved with lexical-semantic representation and retrieval (33, 34). In the non-fluent/agrammatic variant of PPA, we reported associations between WM disruptions in the dorsal stream and grammatical impairments, and we implicated WM damage in the ventral stream in the representation of major lexical grammatical categories (32). Similarly, Wilson et al. correlated damage to dorsal language tracts with impaired syntactic processing in a mixed PPA group (31). The current results augment our knowledge of language processing networks as damage to projections contributing to dorsal and ventral streams in svPPA and lvPPA was related to impaired lexical retrieval.

Our analyses of reduced FA in PPA relative to controls were largely consistent with previous reports. Both the semantic and logopenic variants of PPA were associated with widespread changes in FA. In svPPA, we found evidence of decreased WM fiber integrity in UNC, ILF, SLF, IFO, and CC, but not in CR. FA disruptions were focused in the frontal and temporal portions of the involved structures, with the most extensive involvement of anterior and ventral portions of left-lateralized tracts. Left UNC in particular was the most heavily affected fiber tract. These results correspond closely to the characteristic pattern of anterior temporal GM disease in svPPA (5) and emphasize the breakdown of large-scale neural networks involving multiple GM regions that are important for lexical retrieval in svPPA. Likewise, the WM disruptions in lvPPA provide further evidence of anatomical congruence with GM atrophy and the breakdown of projections between these areas and other GM areas important for lexical retrieval in lvPPA. The focus of GM damage in lvPPA is the posterior temporal lobe and peri-Sylvian regions (6). Visual inspection of results revealed a corresponding posterior shift in WM damage in lvPPA relative to svPPA. Again, FA changes were more prominent in left-lateralized tracts relative to those on the right, and right SLF and UNC were the only tracts where no significant changes in FA were detected. These results are consistent with previous reports that patterns of WM disruptions in PPA variants extend beyond respective anatomic distributions of GM atrophy (13, 35, 36).

In FA studies of svPPA, the profile of WM disease most frequently includes left UNC and ILF (35, 37). Additional studies reporting more widespread alterations include left IFO, left SLF, and the genu of CC (13, 14). One recent study utilizing tract-based spatial statistics [TBSS; Ref. (38)] reported compromised FA in several of these tracts, but results extended into many of the corresponding right-lateralized brain structures (15). The current findings also show that left UNC and ILF were most severely affected, but damage extended into CC, SLF, and even IFO. Further, significant (but less extensive) disease was evident in each corresponding right WM tract. The high sensitivity afforded by the data reduction methods in the TBSS and TSA approaches (16), along with the relatively large groups we studied, may have contributed to the observation of more widespread disease distributions involving right network components. Fewer studies have reported FA disruptions in lvPPA. Previous work has described damage to left SLF and WM of the parietotemporal junction (12, 39). One study reported widespread FA reductions in left WM structures relative to controls (36). Similar to the current results, extensive bilateral WM disease in has been described in lvPPA using TBSS (15).

The TSA approach employed in this study capitalizes on the advantages of three well-established strategies in DTI analysis: fiber tractography, voxel-based morphometry (VBM), and TBSS. Fiber-tracking algorithms facilitate the mapping of statistical features derived from DTI along the length of fiber bundles. Several published techniques extract centerlines from tractography output, normalize these across subjects, and perform statistical inference on DT metrics along the centerlines (40–42). While this approach is apt for fiber bundles that have tubular shapes, such as the cingulum, a centerline representation is inappropriate for other shapes, such as CC. VBM approaches allow for more detailed spatial localization; however, WM structural information is not taken into account during smoothing and analysis (43). TBSS mitigates this concern by incorporating the geometric features of WM structures. In TBSS, a mean FA image is computed, thresholded, and skeletonized; then, the DT metrics of the subjects are projected onto the skeleton for statistical analysis (38). The use of the mean FA image in TBSS limits this approach by ignoring orientation information. Thus, adjacent fasciculi with different orientations but similar FA are combined into a single region of skeleton. Similar to TBSS, TSA utilizes WM structural information and projects diffusion data onto skeleton-based models. However, TSA is structure-specific with connectivity information from fiber tractography incorporated into structural definition. This allows for more reliable inference within anatomical structures. While standard isotropic smoothing in VBM reduces spatial localization comparably in all directions, the geometric modeling employed in TSA allows spatial specificity to be preserved along the more informative directions following the surface of the tract model while being reduced along the depth direction, or the direction orthogonal to the boundary of the structure. Furthermore, the parametric surface representations in TSA allow for the application of surface-based statistical analysis, and robust techniques for the statistical analysis of manifold-based feature maps have been well documented in the context of neuroimaging (16). The patterns of WM tract damage we observed in svPPA and lvPPA both correspond with and extend beyond much of what has been previously observed; these results, benefiting from the advantages of the TSA approach, may represent a more realistic model of WM damage in these PPA variants.

Some limitations in the current study bear review when considering directions for future work. As in other studies assessing DTI within specific PPA variants, the numbers of participating patients were relatively small. Although these groups were larger than those in several previous studies investigating WM in these PPA phenotypes (35, 36, 39), it would be valuable to study larger group sizes to evaluate the TSA technique in a more comprehensive manner. TSA employs data reduction in the direction locally perpendicular to the surface of the tract model. Consequently, some specificity in the localization of results is traded for increased sensitivity to detect changes. However, the directionality of the data reduction maintains a high degree of specificity in the dimensions of greater interest. Also, other studies of WM in PPA have additionally reported MD, trace diffusivity, axial diffusivity, and radial diffusivity (11, 12, 15), but the interpretation of these metrics remains controversial (44, 45). Finally, The tract-specific nature of this method offers a valuable advantage over the TBSS approach by avoiding problems inherent in collapsing adjacent WM tracts in regions of skeleton that have ambiguous directions of diffusion (16). Therefore, improved sensitivity from anatomically guided data reduction in TSA is retained alongside reliable tract-specific localization and robust tensor-based registration. TSA currently incorporates 11 prominent WM tracts whose fibers have widespread projections throughout the brain, but some tracts and regions of WM are not investigated. More complete anatomic coverage would be ideal, although reliable methods of segmentation for several smaller and less easily distinguished WM structures are still an evolving area of research. Furthermore, the canonical set of WM tracts establishes a coordinate system independent of a specific group or study and allows for the possibility of direct comparisons with additional WM studies using the TSA approach.

With these caveats in mind we conclude that distinct patterns of network disruption occur in the semantic and logopenic variants of PPA. Measures of language performance were related to distinct disruptions of WM integrity in svPPA and lvPPA that involve specific structures contributing to a large-scale network for lexical retrieval.

## Authors Contribution

John P. Powers study concept, acquisition of the data, interpretation of the data, statistical analysis, drafting/revising the manuscript for content, final approval of manuscript, agreement to be accountable for all aspects of the work. Corey T. McMillan study concept, interpretation of the data, study supervision and coordination, drafting/revising the manuscript for content, final approval of manuscript, agreement to be accountable for all aspects of the work. Caroline C. Brun study concept, interpretation of the data, statistical analysis, drafting/revising the manuscript for content, final approval of manuscript, agreement to be accountable for all aspects of the work. Paul A. Yushkevich study concept, statistical analysis, revising the manuscript for content, final approval of manuscript, agreement to be accountable for all aspects of the work. Hui Zhang study concept, statistical analysis, revising the manuscript for content, final approval of manuscript, agreement to be accountable for all aspects of the work. James C. Gee study concept, study supervision and coordination, revising the manuscript for content, final approval of manuscript, agreement to be accountable for all aspects of the work. Murray Grossman study concept, interpretation of the data, study supervision and coordination, drafting/revising the manuscript for content, final approval of manuscript, agreement to be accountable for all aspects of the work.

## Conflict of Interest Statement

The authors declare that the research was conducted in the absence of any commercial or financial relationships that could be construed as a potential conflict of interest.

## Supplementary Material

The Supplementary Material for this article can be found online at http://www.frontiersin.org/Journal/10.3389/fneur.2013.00212/abstract

Figure S1 **Gray matter atrophy**. Inflated-brain renderings showing significantly decreased GM density in svPPA **(A)** and lvPPA **(B)** relative to healthy seniors.

## Footnotes

^1^http://dti-tk.sourceforge.net/

^2^http://biomedic.doc.ic.ac.uk/brain-development/
