# Supplementary material for: White Matter Disease Correlates with Lexical Retrieval Deficits in Primary Progressive Aphasia
Source: Front Neurol. 2013 Dec 27;4:212. doi: 10.3389/fneur.2013.00212 (PMC3873600; doi:10.3389/fneur.2013.00212)
Supplement: Figure S1 — Gray matter atrophy. Inflated-brain renderings showing significantly decreased GM density in svPPA (A) and lvPPA (B) relative to healthy seniors. [file 74122_McMillan_DataSheet1.DOCX]

**Supplementary methods**

**Preprocessing of T1-weighted images**

Images were normalized to a standard space and segmented using the PipeDream interface (<http://sourceforge.net/projects/neuropipedream/>) to the ANTS toolkit (<http://www.picsl.upenn.edu/ANTS/>). The ANTS toolkit implements a diffeomorphic and symmetric registration and normalization method that is the most reliable tool available.(1) A local T1 template of 1mm^3^ resolution was built using ANTS from 25 healthy seniors and 25 frontotemporal lobe degeneration patients. Subject images were registered to the local template, the Atropos tool in ANTS(2) used template-based priors to guide three-tissue segmentation (gray matter, white matter, and cerebrospinal fluid), and gray matter (GM) probability images were calculated as a quantitative measure of GM density. GM probability images were then transformed into MNI space for statistical analysis and down-sampled to 2mm^3^ resolution in order to attain a more anatomically relevant voxel size and to decrease the number of comparisons. Finally, images were smoothed in SPM8 (<http://www.fil.ion.ucl.ac.uk/spm/software/spm8>) using a 5-mm full-width half-maximum Gaussian kernel to minimize individual gyral variations. With robust registration and segmentation methods, this conservative smoothing kernel is ideal for detecting focal effects at the level of individual gyri that would otherwise be smoothed across anatomical boundaries.

**Voxel-wise statistical analysis of GM**

We compared GM density between the patient groups and controls with nonparametric, permutation-based statistical analysis using the randomise tool in FSL (<http://fsl.fmrib.ox.ac.uk/fsl/randomise/>). Analyses were run with 10,000 permutations and restricted to voxels containing GM using an explicit mask generated from the average GM probability map of all subjects. Clusters were accepted that survived an extent threshold of 50 voxels and a height threshold of q<0.005 with false discovery rate correction.

**Supplementary figure captions**

**Supplementary figure 1: Gray matter atrophy.** Inflated-brain renderings showing significantly decreased GM density in svPPA (Panel A) and lvPPA (Panel B) relative to healthy seniors.

**Supplementary figure 1**

**Gray matter atrophy**


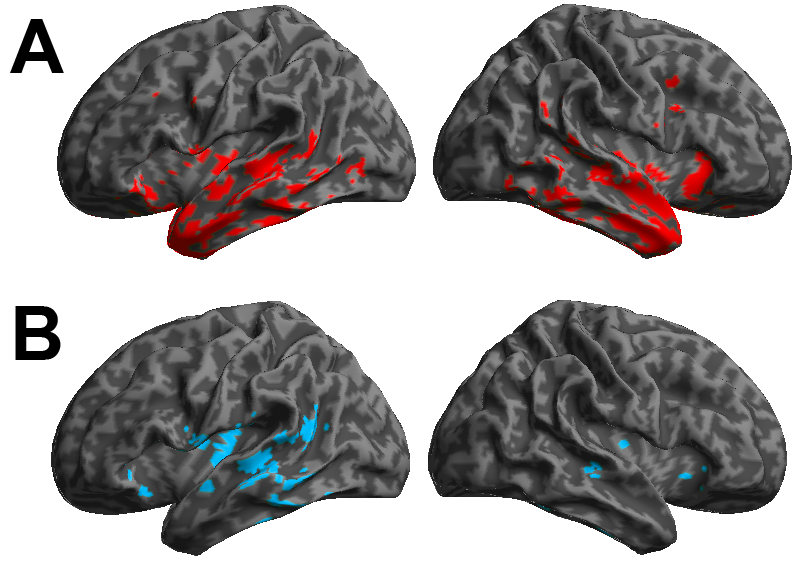


**Supplementary References**

(1) Avants BB, Epstein CL, Grossman M, Gee JC. Symmetric diffeomorphic image registration with cross-correlation: Evaluating automated labeling of elderly and neurodegenerative brain. *Med Image Anal* (2008) **12**: 26-41.

(2) Avants BB, Tustison NJ, Wu J, Cook PA, Gee JC. An open source multivariate framework for n-tissue segmentation with evaluation on public data. *Neuroinformatics* (2011) **9**: 381-400.
